# Endoscopic ultrasound-guided endoscopic submucosal dissection to remove a fishbone embedded adjacent to the thoracic aorta

**DOI:** 10.1055/a-2667-7534

**Published:** 2025-09-04

**Authors:** Yangyang Xiong, Mosang Yu, Chaohui Yu, Zhe Shen

**Affiliations:** 1 71069 Department of Gastroenterology, Zhejiang Provincial Clinical Research Center for Digestive Diseases, The First Affiliated Hospital, Zhejiang University School of Medicine, Hangzhou, China

A 32-year-old woman presented with retrosternal pain after accidentally swallowing a fish bone. Initial laryngoscopy at a local hospital did not detect any foreign body. She was subsequently referred to our institution for further evaluation.

Computed tomography revealed a 16-mm linear high-density foreign body at the distal esophagus, in close proximity to the thoracic aorta ( Fig. 1 ). On gastroscopy, a suspicious submucosal bulge was observed in the distal esophagus, but no foreign body within the lumen ( Fig. 2 **a** ). Endoscopic ultrasound (EUS) subsequently identified a hyperdense echogenic structure within the submucosal layer, confirming that the fish bone was entirely embedded in the esophageal wall ( Fig. 2 **b** ).

**Fig. 1:**
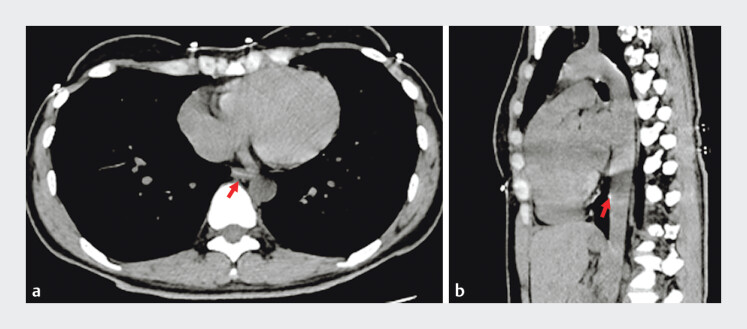
Computed tomography scan revealing the location of the fishbone (arrow). **a** Transverse plane. **b** Sagittal plane.

**Fig. 2:**
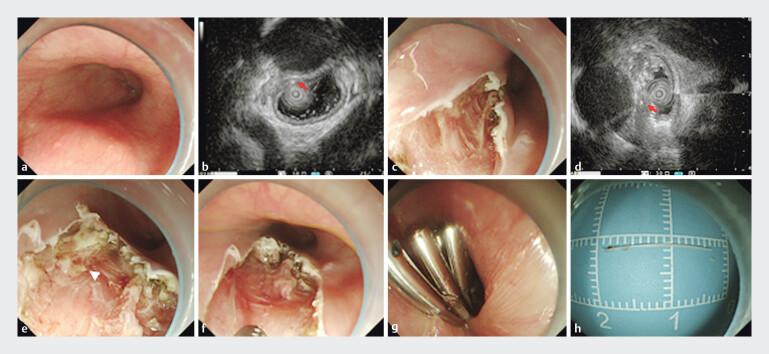
Endoscopic ultrasound (EUS)-guided endoscopic submucosal dissection to remove a fishbone embedded adjacent to the thoracic aorta. **a** On gastroscopy, a suspicious submucosal bulge was observed in the distal esophagus. **b** EUS identified a hyperdense echogenic structure within the submucosal layer of the esophageal wall (arrow). **c** After incision of the submucosal layers at the marked site, no foreign body was found. **d** EUS was repeated to reassess whether the foreign body was located within the muscularis propria. **e** The end of the foreign body was exposed after incision of the circular muscle layer of the muscularis propria (arrow). **f** The foreign body was removed. **g** The wound was closed using titanium clips. **h** The foreign body specimen.

Concerned about surgical risks and complications, the patient opted for a minimally invasive endoscopic approach. Following EUS-guided localization and surface marking, endoscopic submucosal dissection was performed. A disposable mucosal cutting knife was used to incise the mucosa and submucosal layers at the marked site but failed to reveal the foreign body ( Fig. 2 **c** ). EUS guidance was repeated to reassess that the foreign body was located within the muscularis propria ( Fig. 2 **d** ). Subsequently, the circular muscle layer of the muscularis propria was carefully incised, thereby exposing the end of the foreign body ( Fig. 2 **e** ). The fish bone was successfully extracted using foreign body forceps ( Fig. 2 **f** ). No perforation or major bleeding was observed. Finally, the wound was closed completely with titanium clips ( Fig. 2 **g** , Video 1 ). The patient resumed a liquid diet two days after the procedure and was discharged without complications on the third postoperative day.

Endoscopic ultrasound-guided endoscopic submucosal dissection to remove a fishbone embedded adjacent to the thoracic aorta.Video 1

This case presented the successful removal of a foreign body completely embedded in the distal esophageal wall and situated adjacent to the thoracic aorta, using EUS-guided endoscopic submucosal dissection. The most significant difficulty in this case was the anatomical adjacency of the foreign body to the thoracic aorta, which presented a major challenge to safe and effective endoscopic intervention. The key to successful treatment was precise localization of the incision site and depth of incision using EUS, thereby minimizing the risk of secondary injury 1 . In addition, edema and inflammatory changes surrounding the embedded foreign body may obscure the esophageal wall layer architecture on EUS, making precise dissection more challenging 2 . In this context, repeated intraoperative EUS evaluation allowed for dynamic modification of the dissection approach, improving both safety and therapeutic outcomes.

Endoscopy_UCTN_Code_CCL_1AB_2AD_3AB

## Notes

Endoscopy E-Videos https://eref.thieme.de/e-videos
*E-Videos* is an open access online section of the journal *Endoscopy* , reporting on interesting cases and new techniques in gastroenterological endoscopy. All papers include a high-quality video and are published with a Creative Commons CC-BY license. Endoscopy E-Videos qualify for HINARI discounts and waivers and eligibility is automatically checked during the submission process. We grant 100% waivers to articles whose corresponding authors are based in Group A countries and 50% waivers to those who are based in Group B countries as classified by Research4Life (see: https://www.research4life.org/access/eligibility/ ). This section has its own submission website at https://mc.manuscriptcentral.com/e-videos .
